# The Cutaneous Inflammatory Response to Thermal Burn Injury in a Murine Model

**DOI:** 10.3390/ijms20030538

**Published:** 2019-01-28

**Authors:** Zabeen Lateef, Gabriella Stuart, Nicola Jones, Andrew Mercer, Stephen Fleming, Lyn Wise

**Affiliations:** 1 Department of Pharmacology and Toxicology, School of Biomedical Sciences, University of Otago, Dunedin 9054, New Zealand; zabeen.lateef@otago.ac.nz (Z.L.); gabriella.stuart@otago.ac.nz (G.S.); nicky.jones@otago.ac.nz (N.J.); 2 Department of Microbiology and Immunology, School of Biomedical Sciences, University of Otago, Dunedin 9054, New Zealand; andy.mercer@otago.ac.nz (A.M.); stephen.fleming@otago.ac.nz (S.F.)

**Keywords:** thermal burn, inflammation, neutrophil, macrophage, mast cell, Langerhans cell, dendritic cell, collagen, hypertrophic scar, mice

## Abstract

Many burn interventions aim to target the inflammatory response as a means of enhancing healing or limiting hypertrophic scarring. Murine models of human burns have been developed, but the inflammatory response to injury in these models has not been well defined. The aim of this study was to profile inflammatory cell populations and gene expression relative to healing and scarring in a murine model of thermal burns. Cutaneous injuries were created on the dorsal region of C57Bl/6 mice using a heated metal rod. Animals were euthanized at selected time points over ten weeks, with the lesions evaluated using macroscopic measurements, histology, immunofluorescent histochemistry and quantitative PCR. The burn method generated a reproducible, partial-thickness injury that healed within two weeks through both contraction and re-epithelialization, in a manner similar to human burns. The injury caused an immediate increase in pro-inflammatory cytokine and chemokine expression, coinciding with an influx of neutrophils, and the disappearance of Langerhans cells and mast cells. This preceded an influx of dendritic cells and macrophages, a quarter of which displayed an inflammatory (M1) phenotype, with both populations peaking at closure. As with human burns, the residual scar increased in size, epidermal and dermal thickness, and mast cell numbers over 10 weeks, but abnormal collagen I-collagen III ratios, fibre organization and macrophage populations resolved 3–4 weeks after closure. Characterisation of the inflammatory response in this promising murine burn model will assist future studies of burn complications and aid in the preclinical testing of new anti-inflammatory and anti-scarring therapies.

## 1. Introduction

Burns are traumatic injuries that can occur in the home or workplace. There are an estimated 180,000 deaths every year caused by burns, the vast majority in low-middle income countries [1]. With 11 million people requiring medical attention for burns world-wide, in 2004, non-fatal burn injuries represent a leading cause of morbidity. While early burn excision and skin grafting has significantly improved outcomes for these patients, slow healing, infections and scarring still provide major challenges to burn care [2]. As such, burn survivors often have prolonged hospitalization, life-long physical impediments, emotional distress and impaired quality of life.

Thermal and scald burns account for the majority of reported skin burns, with injuries classified as superficial, partial-thickness, full-thickness or subdermal depending on the depth of damage [3]. Superficial burns affect only the epidermis, exhibiting erythema and pain. Partial-thickness burns are classified as superficial or deep, extending to the papillary and reticular dermis, respectively, and can present with blisters, erythema, oedema and diminished sensation. Full-thickness and subdermal burns extend below the skin, and can damage subcutaneous adipose, fascia, muscle or bone. The depth of the burn greatly influences healing outcomes. Superficial burns resolve without scarring within 3 to 5 days. Full-thickness and subdermal burns, however, are slow to heal, require surgical intervention, lead to hypertrophic scarring, and increase the risk of infection, shock and death. Partial-thickness burns are the most common, accounting for 86% of all burns in the recent Bradford Burn Study [4]. While partial-thickness burns are usually non-fatal, those originally deemed to be superficial can progress into deep or full-thickness burns [3]. The wound depth, or area of necrotic tissue, is thought to progress, in part, due to damage to the dermal microvasculature and the resulting tissue hypoxia. Burn wound extension is clinically important as it can confound diagnosis, treatment selection and ultimately patient outcomes.

Each burn type instigates a wound healing response consisting of three over-lapping phases: inflammation, proliferation, and remodelling [5,6]. The response starts with release of histamine, free radicals and inflammatory cytokines, which increase vasodilation and tissue oedema. This brings neutrophils and monocytes to the site, which in turn provide chemotactic signals that recruit macrophages. The inflammatory cells then phagocytose necrotic tissue, protect against pathogens, and produce growth factors that initiate migratory and proliferative responses. Keratinocytes then re-epithelialize the wound, and the vascularized granulation tissue is restored by endothelial cells and fibroblasts. In parallel, fibroblasts differentiate into myofibroblasts, contributing to burn contraction and to deposition and realignment of collagen fibres, in a manner that determines scar pliability.

As burn-associated deaths decrease in high-income countries, the aim of burn care is shifting towards improving complications associated with an impaired healing response [2]. Healing rate correlates with the extent of damage, as deeper burns show a greater inflammatory response. Analyses of human burn tissues indicate that a delay in neutrophil infiltration is associated with increased burn wound depth [7], with deep burn wounds showing peak influx of neutrophils at one week post burn, with a transition to macrophages within 2–3 weeks [8,9,10]. However, in patients with severe burn injuries that cover a greater body surface area, or in patients that fail to survive, this inflammatory cell influx begins later and persists longer [8,9]. Pro-inflammatory cytokine levels also peak after 1–2 weeks in the blood of adults with deep burn wounds [11,12], while increased and sustained cytokine production is associated with more severe burn wounds and with non-survival [13,14]. Damage to the skin barrier also disrupts resident immune cells, with activation and emigration of epidermal Langerhans cells (LC) and dermal dendritic cell (DC) from the skin [15] likely to increase susceptibility to infection, and the risk of sepsis and systemic inflammation. Hypertrophic scarring [16], which arises from deep burn wounds, leads to pain, pruritus and impaired motility, and is characterised by prolonged wound inflammation, contraction, fibrosis, abnormal collagen deposition, and excess LC and mast cell numbers [17,18]. The inflammatory response is therefore central to most burn complications. 

In order to understand burn complications, optimise current treatment regimes and identify novel therapeutic targets, reliable animal models are needed. The choice of a reproducible experimental model is crucial and ideally should be as close as possible to replicate burn injuries in humans. A range of species have been evaluated for burn studies [19,20], and one of the most commonly used is the mouse. With a short healing time, robust immune system, cost effective housing, maintenance and reproduction, and availability of genetically-modified variants and specific reagents, murine models have provided key insights into burn healing response [21,22,23,24]. The mouse does, however, have its limitations as a burn model, as its skin is thin and healing occurs primarily through contraction and not re-epithelialization as in humans [23]. The abundance of hair follicles also provides an enriched pool of progenitor cells that mean healing in mice is accelerated relative to humans [25]. Differences also exist in the immune response between humans and mice, particularly with regards to chemokines, cytokines and cytokine receptors [26,27]. These differences mean that unlike with humans, burn injuries in mice do not lead to excessive scarring [23]. Scarring does appear more hypertrophic following chemical burns [28], bleomycin treatment [29], applied mechanical force [30] and in tight-skin mice [31]. Another issue with murine models is that there has been no standardization with regards to the burn generation technique used. Numerous techniques have been tried, including heated water [22], sodium hydroxide [28], hot air [21] or heated metal [24,28,32], but with varying consistency with regards to burn depth. Arguably, the most consistent method of application is the heated metal rod, where by the burn depth correlates with contact time [33]. But, as with many burn techniques, the inflammatory responses in this model have not been well defined. 

So, to improve the translatability of any findings generated in this murine thermal burn model, key inflammatory processes need to be assessed to identify similarities and differences to human burns. The aim of this study was therefore to create partial-thickness burns in C57Bl/6 mice using a heated metal rod, and to profile inflammatory cell populations and gene expression relative to healing and scarring kinetics over a 10-week period. A partial-thickness wound model was chosen as this burn type represents a significant clinical challenge that requires, and would likely respond to, therapeutic intervention. The burn wound was left open as there is no clear evidence from human clinical trials to support the use of dressing for partial-thickness burns [34]. Changes in wound area, width, cell death and re-epithelialization over time were examined to assess burn severity, and the rate and method of burn closure, relative to that of human burns [19,24,35,36]. The scar area, thickness and collagen content were examined over the 10-week period to assess any commonalities with human hypertrophic scars [29,37,38,39,40]. Changes in blood-derived neutrophils and macrophage and in skin-resident mast cells, DC and LC were evaluated due to their reported presence in human burns or scars [8,9,15,17,18]. Inflammatory cytokines, chemokines, growth factors and collagen subtypes were analysed due to their detection in the blood of human burn patients or reported role in hypertrophic scarring [12,13,41,42,43]. Our hypothesis was that this model would generate a reproducible burn injury characterised by a robust inflammatory response peaking prior to burn contraction and closure like the equivalent human burn, but unlike with human burns, result in minimal scarring.

## 2. Results

### 2.1. Partial-Thickness Thermal Burns Heal through Contraction and Re-epithelialization

**Figure 1:**
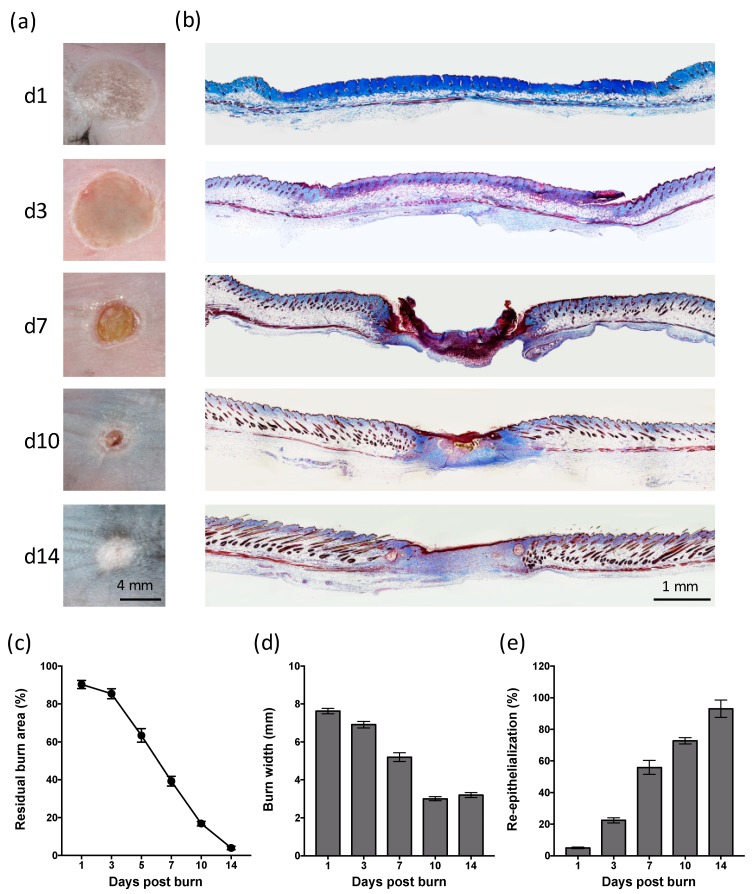
Heated metal rod burns heal through contraction and re-epithelialization. (**a**) Photographs and (**b**) images of Martius, Scarlet and Blue (MSB)-stained sections of healing skin at the indicated day post thermal burn. Scale is as indicated. (**c**) Burn closure is shown as a change in the percentage of the original burn area over-time. (**d**) Burn contraction is shown as the change in burn width over-time. (**e**) Burn re-epithelialization is shown as the change in the percentage neo-epidermal coverage over-time. Data represents the mean ± SEM, *n* = 8.

**Figure 2:**
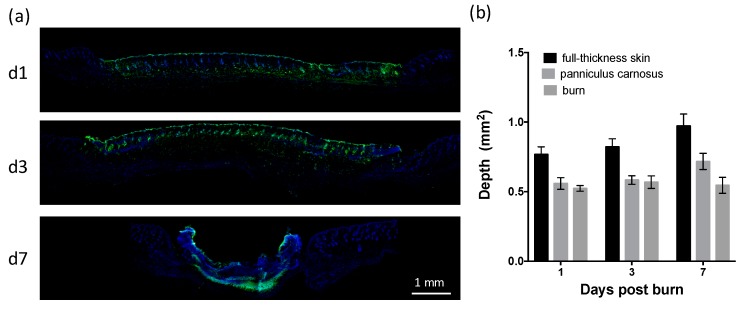
Application of a heated metal rod results in a partial-thickness skin burn. (**a**) Images of healing skin sections with terminal deoxynucleotidyl transferase-mediated deoxyuridine triphosphate nick-end labelling (TUNEL) at the indicated day post thermal burn. Scale is as indicated. (**b**) Burn depth over time is shown relative to the depth of full-thickness skin and the panniculus carnosus. Data represents the mean ± SEM, *n* = 6.

To establish the time course of cutaneous healing in mice following a heated metal rod burn, photographs were taken of the healing skin (Figure 1a), with histological analysis conducted on sections of skin biopsies (Figure 1b). Burns quickly reduced in size from day 3 (Figure 1a,b), with a 10% reduction in burn area per day, and complete closure achieved by day 15 (Figure 1c). Contraction of the burn was evident prior to closure, with a 50% reduction in burn width between day 3 and day 10 (Figure 1d). Re-epithelialization of the burn began at day 3, with ~50% coverage by day 7, and complete coverage achieved by day 14 (Figure 1d). DNA fragmentation characteristic of apoptotic and necrotic cells progressed from the upper dermis, hair follicles, and adipose layer at day 1, to the eschar at day 7 (Figure 2a). The depth of the burn was consistent (0.55 ± 0.04 mm), and equivalent to the depth of the panniculus carnosus (PC) (Figure 2b). These results indicate that this thermal burn produces a consistent partial-thickness injury that heals through both contraction and re-epithelialization within two weeks. 

### 2.2. Partial-Thickness Skin Burns Result in a Persistent Scar

**Figure 3:**
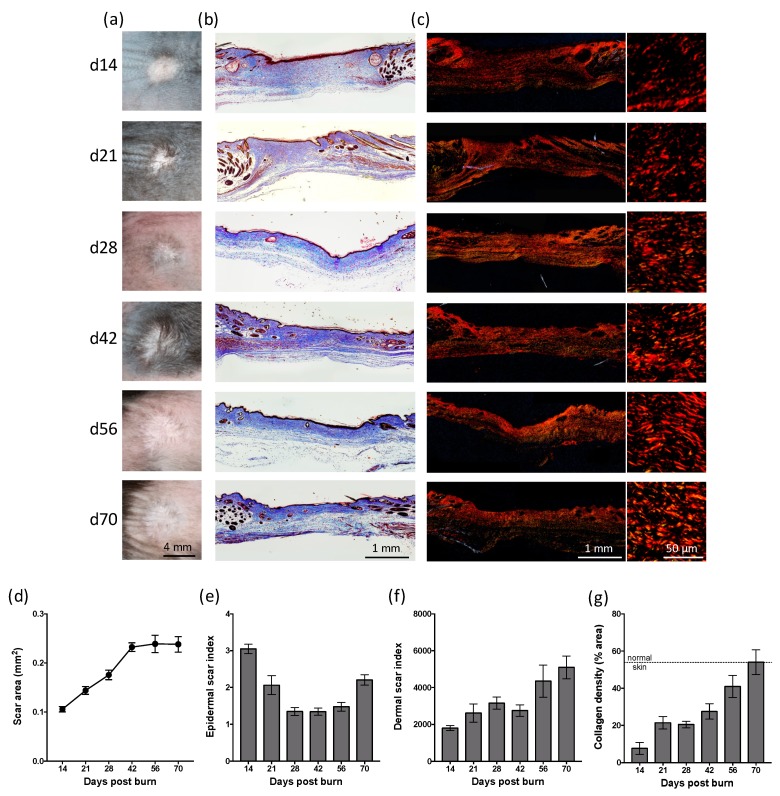
Heated metal rod burns result in a persistent scar. (**a**) Photographs and images of (**b**) MSB-stained and (**c**) picrosirius red-stained sections of skin scars at the indicated day post-thermal burn. Collagen deposition within the scars is shown at higher magnification in the right panel. Scale is as indicated. (**d**) Scar area is shown as a change in the percentage of the area over-time. (**e**) Epidermal scar index is shown as the change in epidermal width within the scar relative to adjacent skin over-time. (**f**) Dermal scar index is shown as the change in the dermal scar area relative to the average scar width over-time. (**g**) Collagen density is shown as the change in the percentage of the area of collagen staining over-time. Data represents the mean ± SEM, *n* = 8.

To establish the extent of scarring in mice following a heated rod burn, photographs were taken of the residual scar at the indicated time points (Figure 3a), with histological analysis conducted on sections of skin biopsies (Figure 3b). The residual scar increased in size from day 14 to day 42 (Figure 3a–c), with a final area ~50% that of the original burn (Figure 3d). The epidermis of the scar was three times that of undamaged skin at day 14 but had returned to normal thickness by day 28 (Figure 3e). At day 70, however the epidermal scar thickness had increased to twice that of undamaged skin (Figure 3e). The dermal scar to increase in area relative to its thickness over the 70 days. (Figure 3f). Collagen density within the scar also increased over time and was equivalent in abundance to undamaged skin by day 70 (Figure 3g). Parallel collagen fibre formation was observed from day 28 to day 56, with basket weave-like texture reminiscent of normal skin evident at day 70 (Figure 3c). There also appeared to be an increase in the presence of skin appendages, such as hair follicles, within the scar at day 70 (Figure 3c). These results indicate that this thermal burn produces a scar that increases in size and matures over 10 weeks.

### 2.3. Partial-Thickness Skin Burns Lead to Changes in the Inflammatory Cell Population during the Healing and Scarring Process

**Figure 4:**
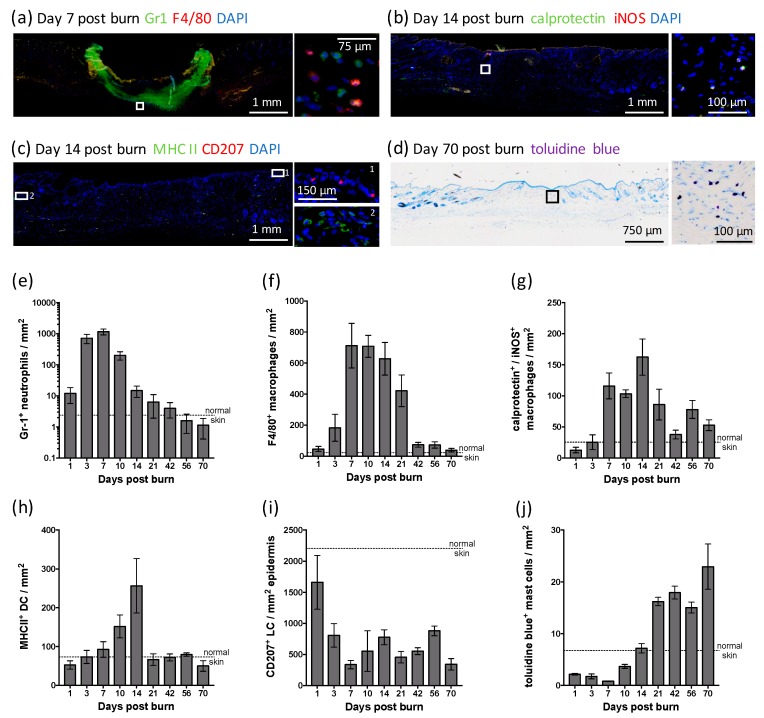
Profile of inflammatory cell populations during healing and scarring of heated rod burns. Images of skin sections stained with 4′,6-diamidino-2-phenylindole (DAPI) and antibodies against (**a**) Gr-1 and F4/80, (**b**) calprotectin and iNOS, (**c**) MHC II and CD207, or with (**d**) toluidine blue at the indicated day post thermal burn. Cell-specific staining is shown at higher magnification in the right panel. Scale is as indicated. Changes in the number of (**e**) Gr-1^+^ neutrophils, (**f**) F4/80^+^ macrophages, (**g**) calprotectin+ and iNOS^+^ macrophages, (**h**) MHC II^+^ DC, (**i**) CD207^+^ LC, and (**j**) toluidine blue^+^ mast cells within the burn/scar over time are expressed relative to area. Values for normal skin are as indicated. Data represents the mean ± SEM, *n* = 6.

To establish inflammatory cell dynamics following a heated rod burn, immunofluorescent and histological analyses were conducted on sections of skin biopsies (Figure 4a–d). Immunofluorescent staining for the neutrophil marker, granulocyte-differentiation antigen (Gr-1), was evident within the burn and scar tissue from day 1 to day 42 (Figure 4e) but was most abundant in the eschar at day 7 (Figure 4a). F4/80 staining showed a substantial influx of macrophages below the burn at day 7 (Figure 4a), persisting at that level in the scar until day 21 and remaining above that of normal skin at all time-points examined (Figure 4f). Staining for calprotectin and inducible nitric oxide synthase (iNOS), which are expressed in M1 macrophages, showed an increase in double-stained cells from day 7 that peaked at day 14 within the scar tissue (Figure 4b), then persisted above that of normal skin (Figure 4g). The markers for antigen presenting cells of the skin—dermal DC and epidermal LC—showed contrasting profiles. Major histocompatibility complex (MHC) class II-positive DC increased within the healing burn, peaking at day 14 (Figure 4c,h), then reducing to the number found in normal skin from day 21. Cluster of differentiation (CD)207-positive LC however decreased over time, remaining at ~20% of that of normal skin from day 7 to day 70 (Figure 4c,i). Toluidine blue staining showed a reduction in skin-resident mast cell granules until day 14, with numbers increasing in the scar tissue to higher than that of normal skin, peaking at day 70 post burn (Figure 4d,j). These results indicate there is a strong influx of neutrophils, DC and macrophages following this thermal burn, with a contrasting reduction in epidermal LC and dermal mast cells. Changes in the inflammatory cell populations also persist during scar remodelling, with the numbers of LC and mast cells lower and higher than normal skin, respectively.

### 2.4. Partial-Thickness Skin Burns Lead to Changes in Inflammatory Gene Expression that Peak during the Healing Process, with Sustained Effects on Fibrotic Gene Expression

**Figure 5:**
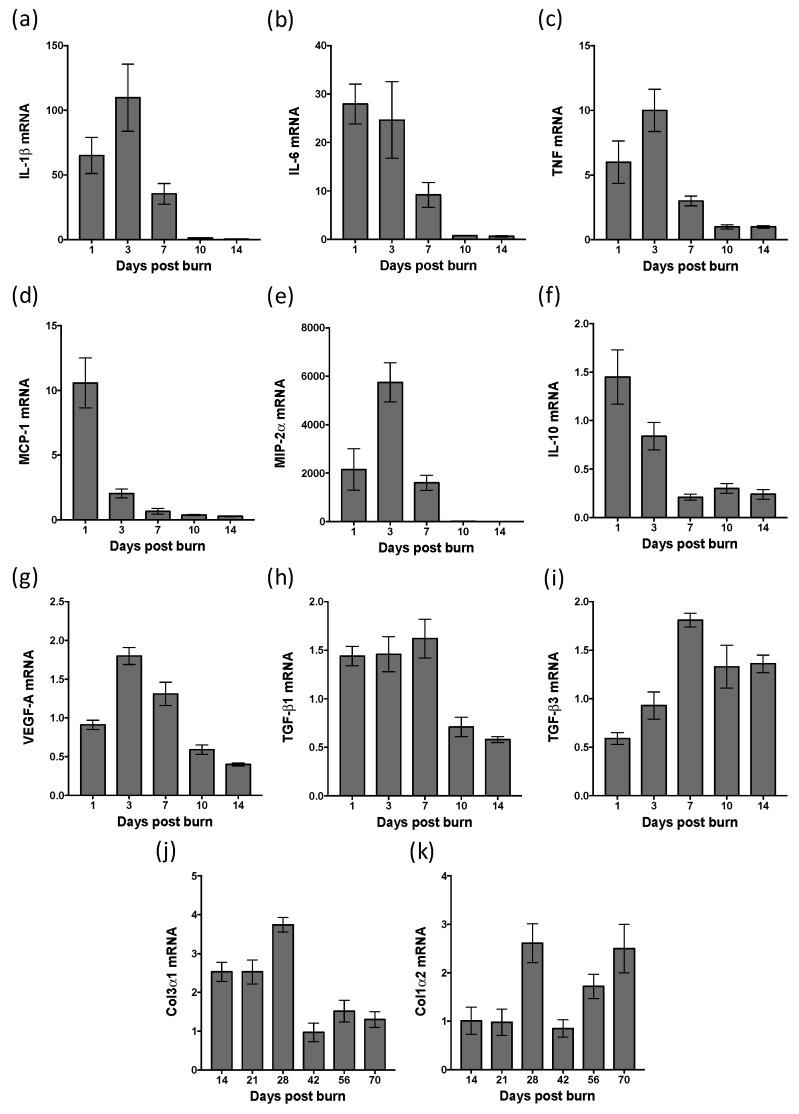
Profile of gene expression during healing and scarring of heated rod burns. Quantitative PCR was used to measure expression of (**a**) IL-1β, (**b**) IL-6, (**c**) TNF, (**d**) MCP-1, (**e**) MIP-2α, (**f**) IL-10, (**g**) VEGF-A, (**h**) TGF-β1, (**i**) TGF-β3 (**j**) Col3α1, and (**k**) Col1α2 in samples taken of healing skin. The level of mRNA is relative to that of TATA box binding protein (TBP) and normal skin. Data represents the mean ± SEM, *n* = 4.

To establish the timing of the inflammatory response following a heated rod burn, quantitative polymerase chain reactions (PCR) were used to profile gene expression in the healing skin (Figure 5). Expression of the pro-inflammatory cytokines, interleukin (IL)-1β, IL-6 and tumour necrosis factor (TNF) peaked in the healing burn at ~day 3, returning to below that of normal skin by day 10 (Figure 5a–c). The pro-inflammatory chemokines, macrophage chemoattractant protein (MCP)-1 and macrophage inflammatory protein (MIP)-2α peaked in burn tissue at day 1 and 3, respectively, again dropping below base-line at day 10 and day 14 (Figure 5d,e). The anti-inflammatory cytokine, IL-10, initially increased in expression within the burn at day 1, but then decreased well below that of undamaged skin from day 7 (Figure 5f). Expression of vascular endothelial growth factor (VEGF)-A, which is critical for vascularization, increased in skin burns only at day 3 and day 7 (Figure 5g). Regulators of fibroblast function, transforming growth factor (TGF)-β1 and TGF-β3, showed contrasting expression patterns, with TGF-β1 expression greater than that of normal skin from day 1 to day 7, while TGF-β3 expression increased from day 7 to day 14 (Figure 5h,i). With each of these genes, the expression level within scar tissue from day 21 to day 70 post burn was equivalent to that of normal skin. Expression of the predominant collagen (Col) types in the skin, Col3α1 and Col1α2, was initially less than that of normal skin but then showed contrasting expression patterns, with Col3α1 expression greater than that of normal skin from day 14 to day 28, and Col1α2 expression increased at day 28 and from day 56 to day 70 (Figure 5j,k). These findings suggest that this thermal burn induces a strong pro-inflammatory response that subsides prior to burn closure, but that leads to sustained effects on scar remodelling.

## 3. Discussion

The inflammatory response is central to most burn complications, and many current and new burn interventions aim to target this process. Reliable animal models are therefore critical to defining to the role of inflammation in the impaired healing, susceptibility to infection and hypertrophic scarring observed post burns in humans. Reproducible murine burn models have been developed, but the inflammatory responses to injury in these models have not been well defined. So, to improve the translatability of any findings generated in this murine thermal burn model, the inflammatory processes need to be assessed, so as to identify key similarities and differences with human burns. This study profiled the inflammatory cell populations and gene expression relative to healing and scarring kinetics following a heated metal rod burn in C57Bl/6 mice. Consistent with our hypothesis, this model generated a reproducible, partial-thickness burn injury, which showed a strong influx of neutrophils with increased pro-inflammatory cytokine and chemokine gene expression that preceded wound contraction, and re-epithelialization. In contrast to our hypothesis, the macrophage and mast cell populations persisted into the remodelling phase, despite inflammatory gene expression subsiding, with the burn injury resulting in a large scar that persisted for at least 10 weeks.

Superficial burns in humans are limited to the epidermis and papillary dermis, result in blister formation and tend to heal quickly [3]. Application of a heated rod in this murine model caused cell death to the depth of the panniculus carnosus, lacked blister formation and took two weeks to heal. This suggests the wound was consistent with that of a deep partial-thickness burn in humans. This model also showed an extended influx of inflammatory cells, with the peak neutrophil infiltration after one week, and macrophages numbers peaking 2–3 weeks post burn. This appears consistent with deep partial- or full-thickness burns in humans that survive their injury [8,9,10]. The immediate post-burn expression of IL-6, IL-10, VEGF-A and MCP-1, in the heated rod model is, in turn, consistent with detection of the same inflammatory mediators in the blood of burns patients [11,12,13,14]. However, the peak expression of TNF and IL-1β, 3 days post burn, differs from human blood where these cytokines continue to be detected for at least 60 days [11,13]. Persistent cytokine levels in the blood could reflect systemic inflammation as opposed to burn site expression. Analysis of systemic cytokine levels would therefore be needed to determine if the murine model accurately replicates the systemic inflammation characteristic of human burn wounds.

Wound macrophages exhibit different phenotypes based on their pathway of activation, and the ratio of these phenotypes alters as a wound matures. During the early inflammatory phase of wound healing in mice, 85% of macrophages have an inflammatory M1 phenotype [44]. The ratio switches as the proliferative phase of healing begins, and now 80% of macrophages have a reparative M2 phenotype [44] M1 macrophages are pro-inflammatory, secreting mediators such as calprotectin, nitric oxide, TNF and IL-6, while M2 macrophages are anti-inflammatory and reparative, producing cytokines like IL-10, and growth factors such as platelet-derived growth factor-β, TGF-β1 and VEGF-A. Depletion of macrophages at the early stage of healing in mice reduces inflammation and delays healing [45], also limiting scarring [46], while macrophage depletion mid healing causes a greater delay in wound closure, with sustained inflammation and edema [45]. M2 macrophages also promote wound repair and can exasperate scarring in mice, as chemical inhibition of the M1-M2 transition with GW2580 suppressed collagen production [47], while transplantation of CD301b-positive M2 macrophages increased fibroblast proliferation and angiogenesis [48]. Profiling of macrophage phenotypes in human skin showed infiltration of M1 macrophages for 2 weeks after traumatic or burn injury, while M2 macrophages peaked after 3–4 weeks in the proliferative hypertrophic scar [49]. The peripheral blood of severely burned patients is also dominated by MCP-1-producing M2b monocytes [50]. In the heated rod model, macrophage numbers increased post burn, peaking after 1–2 weeks, and although the influx of M1 macrophages showed a similar pattern, they generally represented only 25% of the total macrophage population at each time point. This ratio is very different to that reported for full-thickness excisional wounds. The ratio of M1 to M2 macrophages may vary according to mouse strain, wound type or depth, but this finding would require further experimental validation. Staining for additional phenotype-specific markers would ensure the antibodies used are sufficiently sensitive to detect macrophages with the M1 phenotype and also clarify whether monocytes in the blood, and macrophages that persist in the scar tissue are of an M2 phenotype.

Peak inflammatory cytokine and chemokine gene expression coincided with tissue necrosis, mast cell degranulation, and the influx of neutrophils in the heated rod model, but levels diminished during the period of the macrophage influx. IL-10, TGF-β1 and VEGF-A, by contrast were at maximal expression level during the first week post burn and were not as abundant when macrophages were at their maximal. This suggests the relative contribution of macrophages to the production of these genes in this burn model is low, and that alternative methods may be needed to assess the gene expression profiles specific to different macrophage phenotypes. The expression of VEGF-A did, however, coincide with the initiation of burn re-epithelialization, which is consistent with studies in which VEGF-A promoted wound re-epithelialization, through induction of keratinocyte proliferation and migration and expression of matrix metalloproteinases [35]. VEGF-A also induces angiogenesis [51], but the extend of vascularization post burn was not examined in this model. The expression of TGF-β1 preceded contraction of the injured skin, with stabilization of burn width in concordance with the increased expression of TGF-β3. Wound contraction is mediated by myofibroblasts, and their differentiation state, contractility and collagen production are dependent on a high TGF-β1 to TGF-β3 ratio [52,53]. These findings suggest myofibroblast numbers peak by day 10 post burn in this heated rod model, but further validation and comparison with human burn tissues is required.

Human skin contains epidermal LC and dermal DC, which are key inducers of the adaptive immune response upon infection. Burn injuries are highly susceptible to infection and in a human ex vivo burn model, thermal injury induced LC and DC emigration from the skin, with upregulation of MHC II and costimulatory molecules such as CD80, CD86 and CD40 [15]. In the heated-rod model, DC numbers initially reduced in the burn but were replenished within 3 days and continued to increase in number until closure was achieved. This suggests the innate immune response was not compromised in these mice, and that DC contribute to wound healing responses. Indeed, other studies in mice have shown DC repopulate the skin by day 4 post burn, modulating neutrophil-mediated anti-microbial responses [54], and accelerating burn closure through enhanced angiogenesis and TGF-β1 expression [55]. While LC emigration from the burn was also noted in the heated rod model, LC depletion was maintained in the remodeling scar. Human hypertrophic scars in contrast exhibit increased numbers of LCs relative to normal scars and normal skin [18]. The immunological consequence of LC loss and its functional relevance to scar formation post burn are therefore unclear.

Systemic infections following burns are linked to a sustained pro-inflammatory response, compounded by compensatory immunosuppressive responses. While inflammatory cells, including M1 macrophages and DC, persisted in the healing skin after thermal injury, localized production of pro-inflammatory mediators subsided quickly. Dermal DC emigrating from ex vivo human burns [15] and splenic DCs in mice subjected to burn injuries [56] reportedly had impaired T cell-activating capacity. This was linked to expression of immunosuppressive factors, such as IL-10, IL-4, TGF-β and PDGF, all produced during scar remodeling. Sustained changes in circulating immune cells have also been observed in mice following a full-thickness burn injury, with increased IL-10 levels in the blood maintained for 84 days [57]. In this model, only transient expression of the anti-inflammatory factor, IL-10 was observed within the burn. This is, however, consistent with the detection of IL-10 in the blood of humans suffering from less severe full-thickness burns [11,13]. Systemic changes in circulating immune cells or cytokine profile were not assessed, and further investigation is required to determine if immune suppression is a feature of this model.

Mast cells are resident in the skin and completely degranulation following wounding, which leads to a reduction in staining with granule-specific dyes [58]. Consistent with this, a reduction in granulated mast cells was observed post burn in the heated rod model. Mast cells contribute to inflammation, re-epithelialization and angiogenesis through the release of histamine and TNF, and the synthesis of IL-1β, IL-6, keratinocyte growth factor and VEGF [59]. In tissues that heal quickly with less inflammation and scarring, such as the skin of early gestation mice [58], and the oral mucosa of pigs [60], reductions in mast cell number, size and degranulation have been reported. Mast cells also affect fibroblasts, modulating collagen remodelling as opposed to production [61], with higher numbers observed in hypertrophic scars than in normal skin or normal scars across human and animal models [17,62]. The high mast cell numbers in the scar tissue in this burn model, is therefore consistent with that of hypertrophic scars.

Human hypertrophic scars increase in size over a period of 3–6 months, then begin to mature, flattening and softening over at least a year. Developing scars exhibit a thickened dermis and epidermis, pronounced inflammatory infiltrate and an abundance of parallel type III collagen fibres [29,37]. Mature hypertrophic scars display more pronounced dermal thickening, and absence of dermal appendages, collagen whorls and a flattened epidermis [29,39]. In the heated rod model, the residual scars gradually increased in area, dermal thickness, and in the abundance and size of parallel collagen fibres. A thickened epidermis and inflammatory infiltrate, with high type III collagen expression was also evident in the early scar, suggesting that for 3–4 weeks this model is consistent with a developing human hypertrophic scar. After this point, the scar began to mature, with a reduced epidermal thickness and inflammatory infiltrate, and increased collagen type I expression. By 10 weeks, however, the scar showed signs of collagen remodeling into basket-weave fibres and skin appendage regeneration, indicating the scar was diminishing [25,38]. This pattern of development and resolution is consistent with other murine models that require mechanical or chemical intervention to generate the hypertrophic scar [28,29,30].

The heated rod murine model has been [24,28,32], and likely will continue to be, used to investigate interventions aimed at treating the impaired healing, infections and hypertrophic scarring associated with human burns. The findings from this study indicate this model mimics the early inflammatory events post burn in humans, so is appropriate for investigating anti-inflammatory and immune-modulatory therapies, and results gained would likely be clinically translatable. The evidence also suggests this model mimics a developing hypertrophic scar, but its use in this regard is limited as the scar naturally resolves. The model could, however, be used to assess therapeutics aimed at hypertrophic scar prevention as opposed to resolution. Another limitation of this model and the study design is that the burn wound was left open, and as such was more susceptible to infection and to interference by the mouse. Clinical treatment of superficial burns involves covering with dressings, although there is controversy regarding their benefits for partial-thickness or deep burns [34]. Dressings aid moisture retention within the burn, prevent eschar formation and enhance re-epithelialization, as well as facilitate the use of topical treatments [2,16]. The healing and scarring kinetics, and the magnitude and timing of the inflammatory response in this model may therefore differ if the heated rod burns are covered with a dressing.

## 4. Materials and Methods 

### 4.1. Murine Thermal Burn Model

All procedures were performed with approval from the Animal Ethics Committee of the University of Otago (#86/013) with animals sourced and housed in the Hercus-Taieri Resource Unit (Dunedin, New Zealand). C57BL/6 mice (female, 8 weeks of age) were anaesthetized by subcutaneous (SC) injection of ketamine/domitor/atropine (75/1/0.05 mg/kg, respectively), the dorsum shaved, depilated and cleaned with saline. Thermal injuries were performed as previously described [32], by placement of an 8 mm-diameter aluminium rod on the dorsum for 4 s, that had been heated for 5 min in boiling water. Mice were given SC injections of saline to prevent dehydration, bupivacaine (2 mg/kg) for pain relief, and amphoprim (30 mg/kg) to prevent infection. No dressing or topical treatments were applied to the burn wounds. Mice were then revived by SC injection of Antisedan (Zoetis, Auckland, New Zealand) (5 mg/kg). Digital photographs were captured with a ruler aligned next to the burn while mice were immobilised with inhaled isoflurane (1–5%). Mice were euthanized at day 1, 3, 7, 10, 14, 21, 28, 42 and 70, with burns surgically excised and bisected along the medial-lateral axis. One half was fixed in 0.5% zinc salts solution and processed into paraffin wax so that the midpoint of the burn was sectioned and compared between groups. The other half had RNA stabilized in RNAlater solution (Qiagen, Hilden, Germany), with storage at −80 °C.

### 4.2. Histology

Skin sections (4 µm) stained with MSB or Toluidine blue were visualized under bright light. Skin sections (4 µm) stained with picrosirius red were visualized under dark-field with a polarized filter. Images were taken of the section at 10× magnification then converted into panoramas using Photoshop (Adobe Systems). Using ImageJ (http://imagej.nih.gov/ij/), measurements were taken between the burn width, and the proportion covered with neo-epidermis. Re-epithelialization was calculated as a percentage of the burn width covered by neo-epidermis. Four epidermal and dermal thickness measurements were taken within the burn, with two further measurements at a distance of 500 µm on either side of the burn. The scar tissue was outlined and the area measured, with the Dermal Scar Index calculated by dividing the scar area by the average dermal thickness. The Epidermal Scar Index was calculated by dividing the average of the internal epidermis thickness measurements by the average of the distal epidermis thickness measurements. Mast cells were quantitated in images taken of Toluidine blue-stained sections. Images were converted to 8 bit, the entire burn/scar tissue area outlined and measured, and the threshold adjusted to highlight the stained cells which were counted using the particle analysis tool. Collagen density within the scar tissue was examined in images of picrosirius red-stained sections. Images were converted to 8 bit, the scar tissue was outlined and the threshold adjusted automatically to highlight the stained collagen, with measurements taken as a percentage of the total scar area. 

### 4.3. Immunofluorescent Histochemistry

Skin sections (4 µm) were incubated with antibodies to Gr-1 (AlexaFluor^®^488-RB6-8C; eBioscience, San Diego, CA, USA; dilution 1:100), F4/80 (AlexaFluor^®^594-BM8; eBioscience; 1:100), calprotectin (MAC387, Abcam, Cambridge, England, 1:200) with goat anti-mouse IgG H + L (AlexaFluor^®^488; Invitrogen, Carlsbad, CA, USA; 1:500), iNOS (rabbit polyclonal ab3523; Abcam; 1:100) with goat anti-rabbit immunoglobulin G (H + L) (AlexaFluor^®^594; Invitrogen; 1:500), MHC class II IA+IE (FITC-M5/114.15.2; Abcam; 1:400); and supernatant from cells expressing antibodies to CD207 (929F3; 1:5) in conjunction with goat anti-rat IgG2a (AlexaFluor^®^594; Invitrogen; 1:500). The Dead End Fluorometric TUNEL System (Promega, Madison, WI, USA) was used according to manufacturer’s instructions. Nuclei were stained with DAPI (Invitrogen, 75 nM) and slides mounted with SlowFade Gold (Invitrogen). Images were taken of the stained section at 40× magnification then converted into panoramas. To quantify the specific cell types, 0.25 mm × 0.25 mm squares were randomly selected within images taken of antibody-stained burn/scar tissue sections, at both the dermal/epidermal junction (4 per section) and the hypodermis (4 per section). The number of stained cells in each square were counted manually, with the number expressed relative its area. Four burn depth measurements were taken from the epidermal surface to the bottom of the apoptotic/necrotic tissue highlighted by TUNEL staining. Two additional measurements were taken, at a distance of 500 µm on either side of the burn, from the epidermal surface to the panniculus carnosus and to the hypodermis/subcutaneous tissue junction. 

### 4.4. Quantitative PCR

Two burn samples from each animal were combined and homogenized, then total RNA isolated. Synthesis of cDNA was carried out with total RNA, oligo(dT)15, and random hexamer primers using Superscript III (Invitrogen). Quantitative PCR were conducted using Applied Biosystems 7500 Fast PCR machine, PerfeCTa^®^ SYBR^®^ Green FastMix^®^ (Quanta BioSciences, Beverly, MA, USA), and cDNA equivalent to 5ng RNA. Primer pairs were as previously described [35,40,63]. Gene expression was normalized to TBP [64] and to unwounded skin taken from age and sex-matched mice.

## 5. Conclusions

This study demonstrated that a heated metal burn produces a highly reproducible, partial-thickness injury in mice, which mimics key aspects of the inflammatory and hypertrophic scarring responses observed in humans, post burn. The partial-thickness burn wounds generated in this model closed within two weeks through both contraction and re-epithelialization as seen with human burns. Again, consistent with human burns, the injury caused an increase in the expression of pro-inflammatory mediators, an influx of neutrophils, and the disappearance of LC and mast cells. This was followed by an influx of DC and macrophages with both populations peaking at burn closure. As with human burns, the residual scar increased in size, epidermal and dermal thickness, and mast cell numbers over the 10-week period, but unlike human burns the abnormal collagen I-collagen III ratios, fibre organization and macrophage populations resolved 3–4 weeks after the burn closed. The model, therefore, has considerable potential to further understanding of the complex processes implicated in burn healing complications, susceptibility to infection, and the formation of hypertrophic scars. To increase knowledge regarding inflammatory and skin-resident immune cells and their role in re-epithelialization and burn contraction, this model should be utilised prior to burn closure, within two weeks. Also in the 3–4 weeks post closure, this model can be used to investigate immune cell populations involved in scar development and remodelling. The use of this model for this purpose may be limited as it does not appear to mimic the systemic inflammation, immune suppression and persistent scarring associated with human burns. However, by characterising the inflammatory response relative to healing and scarring for the murine model, this research provides critical information that will guide the preclinical evaluation of new and much needed anti-inflammatory and anti-scarring treatments for thermal burns. 

## Abbreviations

CD	Cluster of differentiation
Col	Collagen
DC	Dendritic cell
IL	Interleukin
Gr-1	granulocyte-differentiation antigen
iNOS	inducible nitric oxide synthase
LC	Langerhans cell
M1	Inflammatory macrophage
M2	Reparative macrophage
MCP	Macrophage chemoattractant protein
MHC	Major histocompatibility complex
MIP	Macrophage inflammatory protein
MSB	Martius, Scarlet and Blue
PC	Panniculus carnosus
PCR	Polymerase chain reaction
SC	Subcutaneous
TBP	TATA box binding protein
TGF	Transforming growth factor
TNF	Tumour necrosis factor
TUNEL	Terminal deoxynucleotidyl transferase-mediated deoxyuridine triphosphate nick end labelling
VEGF	Vascular endothelial growth factor
